# Impact of chromosomally encoded resistance mechanisms and transferable β-lactamases on the activity of cefiderocol and innovative β-lactam/β-lactamase inhibitor combinations against *Pseudomonas aeruginosa*

**DOI:** 10.1093/jac/dkae263

**Published:** 2024-07-29

**Authors:** Lucía González-Pinto, Isaac Alonso-García, Tania Blanco-Martín, Pablo Camacho-Zamora, Pablo Arturo Fraile-Ribot, Michelle Outeda-García, Cristina Lasarte-Monterrubio, Paula Guijarro-Sánchez, Romina Maceiras, Bartolome Moya, Carlos Juan, Juan Carlos Vázquez-Ucha, Alejandro Beceiro, Antonio Oliver, Germán Bou, Jorge Arca-Suárez

**Affiliations:** Servicio de Microbiología & Instituto de Investigación Biomédica de A Coruña (INIBIC), Complexo Hospitalario Universitario A Coruña, A Coruña, Spain; Servicio de Microbiología & Instituto de Investigación Biomédica de A Coruña (INIBIC), Complexo Hospitalario Universitario A Coruña, A Coruña, Spain; Servicio de Microbiología & Instituto de Investigación Biomédica de A Coruña (INIBIC), Complexo Hospitalario Universitario A Coruña, A Coruña, Spain; Servicio de Microbiología & Instituto de Investigación Biomédica de A Coruña (INIBIC), Complexo Hospitalario Universitario A Coruña, A Coruña, Spain; Servicio de Microbiología & Instituto de Investigación Sanitaria Illes Balears (IdISBa), Hospital Universitario Son Espases, Palma de Mallorca, Spain; CIBER de Enfermedades Infecciosas (CIBERINFEC), Instituto de Salud Carlos III, Madrid, Spain; Servicio de Microbiología & Instituto de Investigación Biomédica de A Coruña (INIBIC), Complexo Hospitalario Universitario A Coruña, A Coruña, Spain; Servicio de Microbiología & Instituto de Investigación Biomédica de A Coruña (INIBIC), Complexo Hospitalario Universitario A Coruña, A Coruña, Spain; Servicio de Microbiología & Instituto de Investigación Biomédica de A Coruña (INIBIC), Complexo Hospitalario Universitario A Coruña, A Coruña, Spain; Servicio de Microbiología & Instituto de Investigación Biomédica de A Coruña (INIBIC), Complexo Hospitalario Universitario A Coruña, A Coruña, Spain; Servicio de Microbiología & Instituto de Investigación Sanitaria Illes Balears (IdISBa), Hospital Universitario Son Espases, Palma de Mallorca, Spain; CIBER de Enfermedades Infecciosas (CIBERINFEC), Instituto de Salud Carlos III, Madrid, Spain; Servicio de Microbiología & Instituto de Investigación Sanitaria Illes Balears (IdISBa), Hospital Universitario Son Espases, Palma de Mallorca, Spain; CIBER de Enfermedades Infecciosas (CIBERINFEC), Instituto de Salud Carlos III, Madrid, Spain; Servicio de Microbiología & Instituto de Investigación Biomédica de A Coruña (INIBIC), Complexo Hospitalario Universitario A Coruña, A Coruña, Spain; CIBER de Enfermedades Infecciosas (CIBERINFEC), Instituto de Salud Carlos III, Madrid, Spain; Servicio de Microbiología & Instituto de Investigación Biomédica de A Coruña (INIBIC), Complexo Hospitalario Universitario A Coruña, A Coruña, Spain; CIBER de Enfermedades Infecciosas (CIBERINFEC), Instituto de Salud Carlos III, Madrid, Spain; Servicio de Microbiología & Instituto de Investigación Sanitaria Illes Balears (IdISBa), Hospital Universitario Son Espases, Palma de Mallorca, Spain; CIBER de Enfermedades Infecciosas (CIBERINFEC), Instituto de Salud Carlos III, Madrid, Spain; Servicio de Microbiología & Instituto de Investigación Biomédica de A Coruña (INIBIC), Complexo Hospitalario Universitario A Coruña, A Coruña, Spain; CIBER de Enfermedades Infecciosas (CIBERINFEC), Instituto de Salud Carlos III, Madrid, Spain; Servicio de Microbiología & Instituto de Investigación Biomédica de A Coruña (INIBIC), Complexo Hospitalario Universitario A Coruña, A Coruña, Spain; CIBER de Enfermedades Infecciosas (CIBERINFEC), Instituto de Salud Carlos III, Madrid, Spain

## Abstract

**Objectives:**

We aimed to compare the stability of the newly developed β-lactams (cefiderocol) and β-lactam/β-lactamase inhibitor combinations (ceftazidime/avibactam, ceftolozane/tazobactam, aztreonam/avibactam, cefepime/taniborbactam, cefepime/zidebactam, imipenem/relebactam, meropenem/vaborbactam, meropenem/nacubactam and meropenem/xeruborbactam) against the most clinically relevant mechanisms of mutational and transferable β-lactam resistance in *Pseudomonas aeruginosa*.

**Methods:**

We screened a collection of 61 *P. aeruginosa* PAO1 derivatives. Eighteen isolates displayed the most relevant mechanisms of mutational resistance to β-lactams. The other 43 constructs expressed transferable β-lactamases from genes cloned in pUCP-24. MICs were determined by reference broth microdilution.

**Results:**

Cefiderocol and imipenem/relebactam exhibited excellent *in vitro* activity against all of the mutational resistance mechanisms studied. Aztreonam/avibactam, cefepime/taniborbactam, cefepime/zidebactam, meropenem/vaborbactam, meropenem/nacubactam and meropenem/xeruborbactam proved to be more vulnerable to mutational events, especially to overexpression of efflux operons. The agents exhibiting the widest spectrum of activity against transferable β-lactamases were aztreonam/avibactam and cefepime/zidebactam, followed by cefepime/taniborbactam, cefiderocol, meropenem/xeruborbactam and meropenem/nacubactam. However, some MBLs, particularly NDM enzymes, may affect their activity. Combined production of certain enzymes (e.g. NDM-1) with increased MexAB-OprM-mediated efflux and OprD deficiency results in resistance to almost all agents tested, including last options such as aztreonam/avibactam and cefiderocol.

**Conclusions:**

Cefiderocol and new β-lactam/β-lactamase inhibitor combinations show promising and complementary *in vitro* activity against mutational and transferable *P. aeruginosa* β-lactam resistance. However, the combined effects of efflux pumps, OprD deficiency and efficient β-lactamases could still result in the loss of all therapeutic options. Resistance surveillance, judicious use of new agents and continued drug development efforts are encouraged.

## Introduction

The main challenge regarding the treatment of severe *Pseudomonas aeruginosa* infections results from the remarkable ability of this pathogen to compromise the activity of all clinically available β-lactams through the selection of chromosomally encoded resistance mechanisms, the growing prevalence of strains producing ESBLs and carbapenemases and the emergence in hospitals worldwide of epidemic MDR/XDR *P. aeruginosa* clones (also known as high-risk clones).^1,2^ The recent introduction of ceftolozane/tazobactam and ceftazidime/avibactam in the clinical setting has alleviated the urgent need for agents to combat MDR/XDR *P. aeruginosa* strains. However, resistance to these new compounds has been increasingly reported, either due to the selection during treatment of mutations in the catalytic pocket of the intrinsic AmpC enzyme of *P. aeruginosa* (PDC) or due to the production of broad-spectrum β-lactamases from genes acquired by horizontal gene transfer.^3–6^

Newly approved compounds with antipseudomonal activity include cefiderocol, imipenem/relebactam, meropenem/vaborbactam and aztreonam/avibactam. Cefiderocol is a siderophore–cephalosporin conjugate with high antipseudomonal activity. This is owed to its increased internalization via iron uptake pathways (such as the PiuA/PiuC or PirA/PirR systems) and to its high stability against mutational resistance mechanisms and the production of broad-spectrum β-lactamases.^7,8^ Imipenem/relebactam and meropenem/vaborbactam are combinations of a carbapenem with, respectively, an innovative diazabicyclooctane- (DBO-) or a β-lactamase inhibitor derived from boronic acid- (BOR-). Both combinations have potent activity against Class A and C β-lactamases, particularly against key targets such as KPC producers.^9^ Aztreonam/avibactam is active against all types of β-lactamases due to the combination of an MBL-stable β-lactam partner with avibactam, which inactivates all serine-type enzymes that can hydrolyse aztreonam.^10^ New combinations based on a DBO- or BOR-type scaffold with antipseudomonal activity and an ultra-broad spectrum of therapeutic coverage, including MBL producers, are in the late stage of development: cefepime/taniborbactam^11^ and cefepime/zidebactam.^12^ Other inhibitors with promising activity are also under development: nacubactam (a DBO with intrinsic anti-PBP-2 activity)^13^ and xeruborbactam (a broad-spectrum BOR-type inhibitor).^14^ Representative figures of the chemical structures of all these β-lactams and β-lactamase inhibitors are shown in Figure S1 (available as Supplementary data at *JAC* Online).

Inclusion of these treatments in the available antibacterial arsenal may represent a step forward in combating *P. aeruginosa* infections. However, their specific activity against the main resistance mechanisms of *P. aeruginosa* has not yet been thoroughly investigated. We challenged these innovative treatments against a large collection of *P. aeruginosa* laboratory isolates expressing the most relevant mutational and transferable β-lactam resistance mechanisms and combinations of these to precisely identify their role in the treatment of this continuously evolving threat.

## Methods

### Collection of PAO1-derived mutants expressing single or multiple combinations of the most relevant chromosomally encoded β-lactam resistance mechanisms found in *P. aeruginosa*

We screened a collection of 17 previously constructed isogenic PAO1-derived knockout mutants expressing the main chromosomally encoded *P. aeruginosa* β-lactam resistance mechanisms. The collection included (i) single knockouts of the *bla*_PDC_ gene and single, double or triple knockouts of its regulators (*dacB*, *ampD*, *ampDh2*, *ampDh3*, *dacC* and *dacG*), covering a wide range of *bla*_PDC_ expression levels; (ii) single knockout mutants of the *oprD* porin gene or the negative local regulators (*mexR*, *mexZ* and *nfxB*) of the main resistance–nodulation–division efflux operons in *P. aeruginosa* (these latter showing overexpression of *mexAB-oprM*, *mexXY* or *mexCD-oprJ*, respectively); (iii) dual knockout mutants including the main combinations of these mechanisms. The characterization of hyperexpression and/or absence of the deleted genes was determined by RT–PCR or PCR plus Sanger sequencing in previous work.^15–18^ A *P. aeruginosa* transposon mutant of *piuC* from a previously described library^19^ was also included due to its previously associated role in the acquisition of cefiderocol resistance in *P. aeruginosa*.^8^

### Construction of a collection of PAO1-derived isolates expressing the most relevant transferable broad-spectrum β-lactamases found in *P. aeruginosa*

To elucidate the specific impact of the acquired β-lactamases circulating in *P. aeruginosa* on the new agents, we designed a library of 27 isogenic PAO1 recombinant isolates expressing: the most relevant Ambler Class A (*bla*_GES-1_, *bla*_GES-5_, *bla*_CTX-M-9_, *bla*_CTX-M-15_, *bla*_SHV-12_, *bla*_PER-1_, *bla*_VEB-1_, *bla*_KPC-2_, *bla*_KPC-3_, *bla*_KPC-31_ and *bla*_KPC-35_), B (*bla*_VIM-1_, *bla*_VIM-2_, *bla*_IMP-13_, *bla*_IMP-28_, *bla*_NDM-1_ and *bla*_NDM-5_), C (*bla*_CMY-2_, *bla*_FOX-4_ and *bla*_DHA-1_) and D (*bla*_OXA-2_, *bla*_OXA-10_, *bla*_OXA-14_, *bla*_OXA-15_ and *bla*_OXA-48_) β-lactamase variants; and two *bla*_KPC_ variants previously selected *in vitro* through the incubation of 2 KPC-producing *Klebsiella pneumoniae* clinical isolates for consecutive days in 10-mL Mueller–Hinton (MH) tubes containing incremental concentrations of imipenem/relebactam up to their 64 × baseline MIC (KPC-2 N132S and KPC-3 L167R).^20^ Although these variants have not yet been detected in the clinical setting, they have potential interest in the study of imipenem/relebactam resistance mechanisms. Each allele was amplified from a previously sequenced clinical isolate available in our laboratory. PCR products were digested and ligated to pUCP-24 (gentamicin resistance marker), a high-copy number shuttle vector that has been extensively used for the expression of β-lactamases and MIC comparisons in *P. aeruginosa*,^21,22^ and transformed into *Escherichia coli* TG1. Transformants were selected in LB agar containing 10-mg/L gentamicin. Recombinant plasmids were extracted, and insert sequences were checked by PCR and Sanger sequencing. Plasmids were electroporated into PAO1, and transformants were selected in LB agar containing 30-mg/L gentamicin. Plasmids were rechecked by PCR and restriction analysis. To obtain an overall view of the interplay between mutational and transferable resistance mechanisms, eight candidate plasmids carrying genes encoding the most widespread Class A, B and D enzymes in *P. aeruginosa* (*bla*_GES-5_, *bla*_PER-1_, *bla*_KPC-3_, *bla*_VIM-1_, *bla*_IMP-28_, *bla*_NDM-1_, *bla*_OXA-14_ and *bla*_OXA-15_) were also transferred in parallel to the PAO Δ*dacB* Δo*prD* or PAO Δ*mexR* Δ*oprD* dual knockout mutants.

### Antimicrobial susceptibility testing

MICs of ceftazidime, ceftazidime/avibactam, ceftolozane/tazobactam, aztreonam, aztreonam/avibactam, cefepime, cefepime/taniborbactam, cefepime/zidebactam, cefiderocol, imipenem, imipenem/relebactam, meropenem, meropenem/vaborbactam, meropenem/nacubactam and meropenem/xeruborbactam for all isolates were determined in triplicate by reference broth microdilution assays. MICs were determined using CAMHB, except for cefiderocol, for which a specifically prepared iron-depleted CAMHB was used.^23^ Tazobactam, avibactam, taniborbactam and relebactam were tested at a fixed concentration of 4 mg/L, whereas vaborbactam and xeruborbactam were tested at 8 mg/L. Zidebactam and nacubactam were tested at a 1:1 ratio with cefepime and meropenem, respectively. EUCAST v 14.0 clinical breakpoints and guidelines (http://www.eucast.org/clinical_breakpoints/) were used. To define susceptibility or resistance to combinations not yet approved, the *P. aeruginosa* EUCAST clinical breakpoints of their β-lactam partners were used. Reference strains *E. coli* ATCC 25922, *E. coli* NCTC 13353, *K. pneumoniae* ATCC BAA-2814 and *P. aeruginosa* ATCC 27853 were used as controls.

### Statistical analysis

Statistical analysis of the antimicrobial activity of the compounds against the isolates studied was performed using Fisher's exact test with the GraphPad software (v 8.3.0).

## Results

### Impact of mutational resistance

A comparative assessment of the impact of the most relevant *P. aeruginosa* mutational β-lactam resistance mechanisms in the activity of the agents under study is summarized in Table 1. The collection of mutants with chromosomal resistance mechanisms showed MICs at susceptibility levels to most of the tested agents. Meropenem and meropenem-based combinations were active against most of the mutants tested, with the exception of the isolate showing combined *mexAB-oprM* overexpression and OprD deficiency. Cefiderocol and imipenem/relebactam were the most potent agents, with the lowest MICs for the mutants studied. The mutant with an inactivated *piuC* gene showed a 16-fold increase in cefiderocol MIC compared with PAO1, with no effect on the MICs of the other β-lactams, evidencing the specific role of the PiuA/PiuC system in cefiderocol internalization. Aztreonam/avibactam, cefepime/taniborbactam and cefepime/zidebactam were very active against mutants showing *bla*_PDC_ overexpression or OprD deficiency. However, their MICs were significantly affected by up-regulation of the *mexAB-oprM*, *mexXY* or *mexCD-oprJ* efflux operons. Overexpression of *mexAB-oprM* conferred borderline resistance to the three combinations, whereas the effects of *mexXY* and *mexCD-oprJ* overexpression were particularly evident for cefepime/taniborbactam. Vaborbactam, nacubactam and xeruborbactam did not improve the activity of meropenem against any of the mutants with an increased expression of *bla*_PDC_ or efflux operons or an inactivated *oprD* gene. Statistical analysis revealed no significant differences for most of the compounds tested since the effect of the studied mutational mechanisms did not usually result in a change in the clinical categorization.

**Table 1.** Antibiotic susceptibility data for PAO1-derived knockout mutants expressing the most relevant *P. aeruginosa* mutation-driven β-lactam resistance mechanisms

			MIC (mg/L)^a^														
Strain	Genotype	Phenotype^b^	CAZ (R > 8)	C/A (R > 8)	C/T (R > 4)	ATM (R > 16)	A/A (R > 16)	FEP (R > 8)	F/T (R > 8)	F/Z (R > 8)	FDC (R > 2)	IMP (R > 4)	I/R (R > 2)	MEM (R > 8)	M/V (R > 8)	M/N (R > 8)	M/X (R > 8)
PAO1	Wild-type	Wild-type	≤1	≤1	≤0.5	≤4	2	2	2	≤1	≤0.125	1	≤0.125	0.5	≤0.125	0.25	0.25
Δ*ampC*	*ampC* knockout mutant	AmpC deficieny	≤1	≤1	≤0.5	≤4	2	2	2	≤1	≤0.125	0.25	≤0.125	0.25	0.25	0.25	0.25
Δ*ampD*	*ampD* knockout mutant	*ampC* overexpression [↑*ampC* ≈ 50-fold]	8	2	1	≤4	2	8	2	2	≤0.125	1	0.5	1	1	0.5	0.5
Δ*ampD* Δ*ampDh2* Δ*ampDh3*	*ampD-ampDh2-ampDh3* knockout mutant	*ampC* overexpression [↑*ampC* ≈ 1000-fold]	32	2	2	8	≤1	8	1	2	≤0.125	1	0.25	0.5	0.25	0.125	0.25
Δ*dacB*	*dacB* (PBP4) knockout mutant	*ampC* overexpression [↑*ampC* ≈ 50-fold]	16	2	1	8	4	16	2	4	≤0.125	1	0.5	1	1	0.5	0.5
Δ*dacB* Δ*ampD*	*dacB* (PBP4)-*ampD* knockout mutant	*ampC* overexpression [↑*ampC* ≈ 1500-fold]	64	4	2	16	4	32	2	4	≤0.125	1	0.25	1	1	0.5	0.5
Δ*dacB* Δ*dacC*	dual *dacB*-*dacC* (PBP4-PBP5) knockout mutant	*ampC* overexpression [↑*ampC* ≈ 500-fold]	16	≤1	2	8	≤1	16	1	2	≤0.125	0.5	0.25	≤0.125	≤0.125	0.25	≤0.125
Δ*dacB* Δ*dacC* Δ*pbpG*	triple *dacB-dacC-dacG* (PBP4-PBP5-PBP7) knockout mutant	*ampC* overexpression [↑*ampC* ≈ 1200-fold]	16	2	2	16	≤1	8	1	2	≤0.125	0.5	0.25	0.25	0.25	≤0.125	≤0.125
Δ*mexR*	*mexR* knockout mutant	*mexAB-oprM* overexpression [↑*mexB* ≈ 10-fold]	8	4	≤0.5	16	8	8	8	8	0.25	1	0.25	2	1	2	2
Δ*ampD* Δ*mexR*	*ampD-mexR* knockout mutant	*ampC* overexpression [↑*ampC* ≈ 50-fold] + *mexAB-oprM* overexpression [↑*mexB* ≈ 10-fold]	32	4	2	16	8	16	8	8	≤0.125	1	0.25	2	2	1	2
Δ*mexZ*	*mexZ* knockout mutant	*mexXY* overexpression [↑*mexY* ≈ 15-fold]	2	≤1	1	≤4	2	8	8	4	≤0.125	1	0.25	0.25	0.25	0.25	0.25
Δ*nfxB*	*nfxB* knockout mutant	*mexCD-oprJ* overexpression [↑*mexD* ≈ 150-fold]	≤1	2	1	≤4	2	8	8	2	≤0.125	1	0.25	0.25	≤0.125	0.25	0.25
Δ*oprD*	*oprD* knockout mutant	OprD deficiency	≤1	≤1	≤0.5	≤4	≤1	2	2	≤1	≤0.125	8	0.25	4	2	2	2
Δ*ampC* Δ*oprD*	dual *ampC*-*oprD* knockout mutant	AmpC deficiency + OprD deficiency	≤1	≤1	≤0.5	≤4	≤1	2	2	≤1	≤0.125	0.25	≤0.125	2	2	2	2
Δ*ampD* Δ*oprD*	dual *ampD*-*oprD* knockout mutant	*ampC* overexpression [↑*ampC* ≈ 50-fold] + OprD deficiency	16	≤1	1	8	2	4	2	2	≤0.125	16	1	4	2	2	4
Δ*dacB* Δ*oprD*	dual *dacB*-*oprD* knockout mutant	*ampC* overexpression [↑*ampC* ≈ 50-fold] + OprD deficiency	32	2	2	8	2	16	2	4	≤0.125	8	1	4	4	4	4
Δ*mexR* Δ*oprD*	dual *mexR*-*oprD* knockout mutant	*mexAB-oprM* overexpression [↑*mexB* ≈ 10-fold] + OprD deficiency	8	2	1	16	8	8	8	4	0.25	16	1	32	16	16	16
Δ*mexZ* ΔoprD	dual *mexZ*-*oprD* knockout mutant	*mexXY* overexpression [↑*mexY* ≈ 15-fold]+ OprD deficiency	≤1	≤1	1	≤4	2	8	8	4	≤0.125	16	1	4	4	2	2
Δ*piuC*	*piuC* transposon-mutant	Impaired iron uptake	≤1	≤1	≤0.5	≤4	2	2	2	≤1	2	1	≤0.125	0.5	≤0.125	0.25	0.25

CAZ, ceftazidime; C/A, ceftazidime/avibactam; C/T, ceftolozane/tazobactam; ATM, aztreonam; A/A, aztreonam/avibactam; FEP, cefepime; F/T, cefepime/taniborbactam; F/Z, cefepime/zidebactam; FDC, cefiderocol; IMP, imipenem; I/R, imipenem/relebactam; MEM, meropenem; M/V, meropenem/vaborbactam; M/N, meropenem/nacubactam; M/X, meropenem/xeruborbactam.

^a^EUCAST v 14.0 breakpoints indicated.

^b^Expression levels were calculated relative to PAO1 in previous work.^15–18^

### Impact of transferable β-lactamases

Comparative MIC data for the 27 PAO1 transformants expressing different β-lactamases are shown in Table 2. Aztreonam/avibactam and cefepime/zidebactam demonstrated the broadest spectrum of activity, with all strains encoding a β-lactamase susceptible to both combinations. The addition of taniborbactam decreased the cefepime MICs to below the resistance breakpoint (*P* < 0.0001) for all strains except for those encoding SHV-12, VIM-1, IMP or NDM enzymes. Cefiderocol was active against transformants harbouring a wide array of β-lactamases, but borderline susceptibility or clinical resistance was noted for those producing SHV-12, PER-1, VEB-1, ceftazidime/avibactam-resistant KPC variants (KPC-31 and KPC-35), NDM enzymes and the extended-spectrum oxacillinases OXA-14 and OXA-15. Imipenem/relebactam was active against transformants carrying most Class A ESBLs, KPC carbapenemases and Class C and D enzymes with cephalosporinase activity but was not active against the KPC-2 N132S and KPC-3 L167R variants, MBLs or OXA-48. None of the meropenem-based combinations significantly potentiated the activity of the carbapenem alone (*P* > 0.05). Meropenem/vaborbactam and meropenem/nacubactam were active against ESBLs and KPC enzymes, whereas xeruborbactam proved to be the most potent inhibitor against the transformants producing GES-5, KPCs or OXA-48. None of these inhibitors potentiated the activity of meropenem against MBL producers. Altogether, when comparing the susceptibility rates of cefiderocol and the new β-lactam/β-lactamase inhibitor combinations with that of ceftazidime/avibactam and ceftolozane/tazobactam, statistically significant higher activity was observed for aztreonam/avibactam, cefepime/taniborbactam, cefepime/zidebactam, cefiderocol, meropenem/nacubactam and meropenem/xeruborbactam (*P* < 0.05).

**Table 2.** Antibiotic susceptibility data of PAO1-derived recombinant isolates expressing the transferable β-lactamases commonly encountered in *P. aeruginosa*

			MIC (mg/L)^a^														
Strain	Ambler Class	Phenotype	CAZ (R > 8)	C/A (R > 8)	C/T (R > 4)	ATM (R > 16)	A/A (R > 16)	FEP (R > 8)	F/T (R > 8)	F/Z (R > 8)	FDC (R > 2)	IMP (R > 4)	I/R (R > 2)	MEM (R > 8)	M/V (R > 8)	M/N (R > 8)	M/X (R > 8)
PAO1	—	Wild-type	≤1	≤1	≤0.5	≤4	2	2	2	≤1	≤0.125	1	≤0.125	0.5	≤0.125	0.25	0.25
GES-1	A	ESBL	32	8	16	≤4	4	16	2	2	0.25	1	0.5	0.5	0.25	0.5	0.25
GES-5	A	Carbapenemase	4	≤1	2	≤4	4	4	2	2	≤0.125	2	0.5	4	2	1	0.25
CTX-M-9	A	ESBL	4	≤1	1	16	2	512	2	8	≤0.125	1	0.25	1	1	0.5	0.25
CTX-M-15	A	ESBL	32	≤1	2	128	2	512	2	4	0.5	1	0.25	0.5	0.5	0.5	0.25
SHV-12	A	ESBL	>512	16	128	>512	16	>512	32	8	8	1	0.25	2	1	1	1
PER-1	A	ESBL	512	32	>256	>512	16	128	2	4	2	1	0.5	0.5	0.5	0.5	0.25
VEB-1	A	ESBL	>512	32	>256	>512	8	256	4	4	2	1	0.5	0.5	0.5	0.5	0.5
KPC-2	A	Carbapenemase	>512	4	>256	512	4	512	2	8	0.5	64	1	64	4	2	0.25
KPC-3	A	Carbapenemase	>512	4	>256	>512	4	512	2	4	1	64	1	64	4	2	0.25
KPC-31^b^	A	ESBL	>512	512	>256	256	4	256	4	8	8	1	0.5	2	1	1	0.25
KPC-35^c^	A	ESBL	>512	32	>256	64	4	256	4	4	4	1	0.5	2	1	1	0.25
KPC-2 N132S	A	Carbapenemase	4	≤1	2	256	4	2	2	2	≤0.125	>128	16	128	32	8	0.25
KPC-3 L167R	A	Carbapenemase	>512	16	>256	256	4	512	4	4	1	>128	8	32	2	4	0.5
VIM-1	B	Carbapenemase	>512	>512	>256	≤4	4	>512	64	4	2	16	8	32	32	32	32
VIM-2	B	Carbapenemase	128	64	>256	≤4	4	64	2	4	0.25	8	8	16	16	16	16
IMP-13	B	Carbapenemase	>512	512	>256	≤4	4	256	256	8	0.5	8	8	16	16	16	16
IMP-28	B	Carbapenemase	>512	>512	>256	≤4	4	>512	512	8	0.5	8	8	64	32	32	64
NDM-1	B	Carbapenemase	>512	>512	>256	≤4	4	>512	32	8	8	32	32	>128	>128	>32	>128
NDM-5	B	Carbapenemase	>512	>512	>256	≤4	2	>512	32	8	8	32	32	>128	>128	>32	>128
CMY-2	C	Extended-spectrum cephamycinase	512	4	64	128	4	128	8	8	1	2	0.25	4	2	2	1
DHA-1	C	Extended-spectrum cephamycinase	256	≤1	32	32	2	8	2	4	≤0.125	1	0.5	1	0.5	0.5	0.25
FOX-4	C	Extended-spectrum cephamycinase	256	32	16	16	8	32	2	4	0.25	1	0.5	1	1	1	0.5
OXA-2	D	Narrow-spectrum oxacillinase	64	4	2	64	2	8	2	2	1	4	1	8	8	4	2
OXA-10	D	Narrow-spectrum oxacillinase	2	≤1	2	32	2	32	2	2	0.5	2	0.5	8	2	2	0.5
OXA-14^d^	D	ESBL	512	256	128	32	8	64	4	4	2	2	0.5	4	2	2	2
OXA-15^e^	D	ESBL	256	64	128	16	2	16	4	4	4	2	0.25	1	0.5	1	1
OXA-48	D	Carbapenemase	≤1	≤1	1	≤4	4	8	2	4	≤0.125	16	8	64	32	16	0.5

CAZ, ceftazidime; C/A, ceftazidime/avibactam; C/T, ceftolozane/tazobactam; ATM, aztreonam; A/A, aztreonam/avibactam; FEP, cefepime; F/T, cefepime/taniborbactam; F/Z, cefepime/zidebactam; FDC, cefiderocol; IMP, imipenem; I/R, imipenem/relebactam; MEM, meropenem; M/V, meropenem/vaborbactam; M/N, meropenem/nacubactam; M/X, meropenem/xeruborbactam.

^a^EUCAST v 14.0 breakpoints indicated.

^b^KPC-31 is a D179Y variant of KPC-3.

^c^KPC-35 is a L169P variant of KPC-2.

^d^OXA-14 is a G157D variant of OXA-10.

^e^OXA-15 is a D149G variant of OXA-2.

### Interplay between mutational and transferable β-lactam resistance

The MIC data for the eight dual PAO *ΔdacB* Δ*oprD* and eight dual PAO Δ*mexR* Δ*oprD* knockout mutants expressing the most widespread ESBLs and carbapenemases are summarized in Tables S1 and S2 (available as Supplementary data at *JAC* Online). The association between the expression of transferable β-lactamases and *bla*_PDC_ or *mexAB-oprM* overexpression with *oprD* inactivation limited the activity of almost all these new compounds, as they were inactive against most of the transformants.

## Discussion

Consistent with data from large-scale surveillance studies,^24^ our findings revealed that cefiderocol and imipenem/relebactam were the most stable agents against *P. aeruginosa* mutational mechanisms. In accordance with recent reports,^8^ inactivation of *piuC* conferred a 16-fold increase in the baseline cefiderocol MIC, providing evidence of the major role that the PiuA/PiuC system plays in cefiderocol internalization (but not in the other β-lactams). Our results also provided interesting data about the effect of efflux on new compounds. Probably the most concerning finding was the effect of the overexpression of the *mexAB-oprM* efflux operon since mutations inactivating *mexR* can be selected during therapy with different β-lactams and result in increased aztreonam/avibactam, cefepime/taniborbactam and cefepime/zidebactam MICs.^25,26^ Finally, our findings revealed that the novel meropenem-based combinations are not expected to play a prominent role against this pathogen, given that meropenem resistance in *P. aeruginosa* is mainly associated with carbapenemase-independent mechanisms.^2^

Regarding transferable β-lactam resistance, aztreonam/avibactam and cefepime/zidebactam outperformed the other combinations (none of the transformants were found to be resistant). Thus, although their activity may be limited to some extent by efflux systems, they are expected to expand the available options to overcome transferable β-lactam resistance in clinical *P. aeruginosa* strains. These considerations could also be extended to cefepime/taniborbactam, which was active against isolates with transferable resistance mechanisms but did not cover IMP-type enzymes. In this regard, recent findings demonstrated that cefepime/taniborbactam-resistant *P. aeruginosa* isolates usually produce IMP-type enzymes or accumulate multiple chromosomal mutations.^26^ In contrast to mutants with chromosomal resistance mechanisms, the effectiveness of cefiderocol was significantly affected by some of the transferable mechanisms analysed here. In accordance with previous findings,^22^ ESBLs, KPCs, NDMs and the Class D OXA-14 and OXA-15 enzymes had a significant impact on the cefiderocol MICs. Given that the genes encoding these enzymes are increasingly reported in *P. aeruginosa* strains worldwide,^1^ close monitoring of their spread is encouraged to extend the lifespan of cefiderocol.

Imipenem/relebactam was not active against transformants producing an MBL or OXA-48, as previously observed.^24^ This combination was also inactive against the transformants harbouring the variants recently described *in vitro* KPC-2 N132S and KPC-3 L167R, which have shown resistance to relebactam inhibition in terms of *K*_iapp_ and IC_50_.^20^ On the other hand, the poor performance of meropenem-based combinations against most of these transformants could be explained by the following: (i) the poor inhibitory potency of vaborbactam against most of the carbapenemases included in the collection (its activity is restricted to KPC enzymes)^9^; (ii) the low intrinsic activity of nacubactam against *P. aeruginosa*, owing to the higher intrinsic resistance of this pathogen (MICs of 32 mg/L when tested alone)^13^; (iii) the poor performance of xeruborbactam against most of the MBLs included (despite its previously observed efficacy in Enterobacterales), which has recently been associated to the activity of the MexAB-OprM efflux pump in *P. aeruginosa*.^27^ Finally, the interplay between mutational and transferable resistance resulted in challenging phenotypes against which almost none of the options evaluated here was active. Altogether, our findings reveal the complexity of developing new antipseudomonal β-lactam-based therapies and highlight the yet unresolved therapeutic gaps regarding this continuously evolving pathogen.

## Supplementary Material

dkae263_Supplementary_Data

## Transparency declarations

Merck Sharp & Dohme (MSD) provided relebactam powder and did not exercise any control over the conduct or reporting of the research. Shionogi provided cefiderocol powder and did not exercise any control over the conduct or reporting of the research. J.C.V.-U. has received honoraria for lectures and/or presentations from MSD. A.O. has received grants or contracts from MSD, Wockhardt and Shionogi and consulting fees and honoraria for lectures and/or presentations from MSD, Pfizer and Shionogi. G.B. has received funding and study materials from MSD, grants or contracts from MSD, Pfizer, ABAC Therapeutics and Roche, consulting fees and honoraria for lectures and/or presentations from MSD, Shionogi, Pfizer, Roche and Menarini, and support for attending meetings and/or travels from Pfizer. J.A.-S. has received honoraria for lectures and/or presentations from Shionogi and Advanz Pharma. All other authors: none to declare.

## Supplementary data

Figure S1 and Table S1 and S2 are available as Supplementary data at *JAC* Online.
