# Supplementary material for: Impact of chromosomally encoded resistance mechanisms and transferable β-lactamases on the activity of cefiderocol and innovative β-lactam/β-lactamase inhibitor combinations against Pseudomonas aeruginosa
Source: J Antimicrob Chemother. 2024 Jul 29;79(10):2591–7. doi: 10.1093/jac/dkae263 (PMC11441999; doi:10.1093/jac/dkae263)
Supplement: dkae263_Supplementary_Data [file dkae263_supplementary_data.docx]

**SUPPLEMENTARY DATA**

**Figure S1.** Detailed comparative view of the chemical structure of the innovative β-lactam antibiotics and β-lactamase inhibitors used in this study. β-lactamase inhibitors are grouped according to their chemical scaffold.

| **Table S1.** Antibiotic susceptibility data of the PAO1 Δ*dacB* Δ*oprD* dual knockout mutant expressing the most relevant ESBLs and carbapenemases found in *P. aeruginosa.* | | | | | | | | | | | | | | | | | | |
| --- | --- | --- | --- | --- | --- | --- | --- | --- | --- | --- | --- | --- | --- | --- | --- | --- | --- | --- |
|  |  |  |  | MIC (mg/L) ^a^ | | | | | | | | | | | | | | |
| Strain | Ambler class | Genotype | Phenotype^b^ | CAZ  (R>8) | C/A  (R>8) | C/T  (R>4) | ATM  (R>16) | A/A  (R>16) | FEP  (R>8) | F/T  (R>8) | F/Z  (R>8) | FDC  (R>2) | IMP  (R>4) | I/R  (R>2) | MEM  (R>8) | M/V  (R>8) | M/N  (R>8) | M/X  (R>8) |
| PAO1 | - | Wild-type | Wild-type | ≤1 | ≤1 | ≤0.5 | ≤4 | 2 | 2 | 2 | ≤1 | ≤0.125 | 1 | ≤0.125 | 0.5 | ≤0.125 | 0.25 | 0.25 |
| PAO1 Δ*dacB* Δ*oprD* |  | dual *dacB*-*oprD* knockout mutant | *ampC* overexpression [↑*ampC* ≈ 50-fold] + OprD deficiency | 32 | 2 | 2 | 8 | 2 | 16 | 2 | 4 | ≤0.125 | 8 | 1 | 4 | 4 | 4 | 4 |
| GES-5 | A | dual *dacB*-*oprD* knockout mutant + *bla*_GES-5_ | *ampC* overexpression [↑*ampC* ≈ 50-fold] + OprD deficiency + Carbapenemase | 32 | 2 | 4 | 16 | 4 | 32 | 4 | 4 | 0.25 | 32 | 8 | 256 | 128 | 8 | 4 |
| PER-1 |  | dual *dacB*-*oprD* knockout mutant + *bla*_PER-1_ | *ampC* overexpression [↑*ampC* ≈ 50-fold] + OprD deficiency + ESBL | >512 | 32 | >256 | >512 | 32 | 512 | 4 | 8 | 4 | 16 | 2 | 4 | 4 | 2 | 2 |
| KPC-3 |  | dual *dacB*-*oprD* knockout mutant + *bla*_KPC-3_ | *ampC* overexpression [↑*ampC* ≈ 50-fold] + OprD deficiency + Carbapenemase | >512 | 8 | >256 | >512 | 4 | >512 | 4 | 8 | 1 | >256 | 16 | 512 | 64 | 8 | 4 |
| VIM-1 | B | dual *dacB*-*oprD* knockout mutant + *bla*_VIM-1_ | *ampC* overexpression [↑*ampC* ≈ 50-fold] + OprD deficiency + Carbapenemase | >512 | 512 | >256 | 16 | 4 | >512 | 256 | 4 | 1 | 256 | 256 | 128 | 128 | 64 | 64 |
| IMP-28 |  | dual *dacB*-*oprD* knockout mutant + *bla*_IMP-28_ | *ampC* overexpression [↑*ampC* ≈ 50-fold] + OprD deficiency + Carbapenemase | >512 | >512 | >256 | 8 | 4 | 512 | 256 | 4 | 0.5 | 64 | 64 | 128 | 128 | 128 | 128 |
| NDM-1 |  | dual *dacB*-*oprD* knockout mutant + *bla*_NDM-1_ | *ampC* overexpression [↑*ampC* ≈ 50-fold] + OprD deficiency + Carbapenemase | >512 | >512 | >256 | 16 | 4 | >512 | 256 | 4 | 16 | 256 | 256 | 512 | 512 | 128 | 256 |
| OXA-14 ^c^ | D | dual *dacB*-*oprD* knockout mutant + *bla*_OXA-14_ | *ampC* overexpression [↑*ampC* ≈ 50-fold] + OprD deficiency + ESBL | 512 | 128 | 64 | 32 | 8 | 32 | 4 | 4 | 2 | 16 | 4 | 16 | 16 | 16 | 8 |
| OXA-15 ^d^ |  | dual *dacB*-*oprD* knockout mutant + *bla*_OXA-15_ | *ampC* overexpression [↑*ampC* ≈ 50-fold] + OprD deficiency + ESBL | 256 | 128 | 256 | 16 | 4 | 32 | 16 | 8 | 4 | 8 | 1 | 16 | 16 | 16 | 8 |
| CAZ: ceftazidime; C/A: ceftazidime/avibactam; C/T: ceftolozane/tazobactam; ATM: aztreonam; A/A: aztreonam/avibactam; FEP: cefepime; F/T: cefepime/taniborbactam; F/Z: cefepime/zidebactam; FDC: cefiderocol; IMP: imipenem; I/R: imipenem/relebactam; MEM: meropenem; M/V: meropenem/vaborbactam; M/N: meropenem/nacubactam; M/X: meropenem/xeruborbactam.  ^a^ EUCAST v 14.0 breakpoints indicated.  ^b^ Expression levels were calculated relative to PAO1 in previous work.^15–18^.  ^c^ OXA-14 is a G157D variant of OXA-10.  ^d^ OXA-15 is a G149R variant of OXA-2. | | | | | | | | | | | | | | | | | | |

| **Table S2.** Antibiotic susceptibility data of the PAO1 Δ*mexR* Δ*oprD* dual knockout mutant expressing the most relevant ESBLs and carbapenemases found in *P. aeruginosa.* | | | | | | | | | | | | | | | | | | |
| --- | --- | --- | --- | --- | --- | --- | --- | --- | --- | --- | --- | --- | --- | --- | --- | --- | --- | --- |
|  |  |  |  | MIC (mg/L) ^a^ | | | | | | | | | | | | | | |
| Strain | Ambler class | Genotype | Phenotype ^b^ | CAZ  (R>8) | C/A  (R>8) | C/T  (R>4) | ATM  (R>16) | A/A  (R>16) | FEP  (R>8) | F/T  (R>8) | F/Z  (R>8) | FDC  (R>2) | IMP  (R>4) | I/R  (R>2) | MEM  (R>8) | M/V  (R>8) | M/N  (R>8) | M/X  (R>8) |
| PAO1 | - |  | Wild-type | ≤1 | ≤1 | ≤0.5 | ≤4 | 2 | 2 | 2 | ≤1 | ≤0.125 | 1 | ≤0.125 | 0.5 | ≤0.125 | 0.25 | 0.25 |
| PAO1 Δ*mexR* Δ*oprD* |  | dual *mexR*-*oprD* knockout mutant | *mexAB-oprM* overexpression [↑*mexB* ≈ 10-fold] + OprD deficiency | 8 | 2 | 1 | 16 | 8 | 8 | 8 | 4 | 0.25 | 16 | 1 | 32 | 16 | 16 | 16 |
| GES-5 | A | dual *mexR*-*oprD* knockout mutant + *bla*_GES-5_ | *mexAB-oprM* overexpression [↑*mexB* ≈ 10-fold] + OprD deficiency + Carbapenemase | 16 | 8 | 2 | 16 | 16 | 16 | 8 | 8 | 0.25 | 32 | 8 | 64 | 32 | 16 | 16 |
| PER-1 |  | dual *mexR*-*oprD* knockout mutant + *bla*_PER-1_ | *mexAB-oprM* overexpression [↑*mexB* ≈ 10-fold] + OprD deficiency + ESBL | >512 | 256 | >256 | >512 | 128 | 512 | 16 | 8 | 8 | 32 | 4 | 16 | 16 | 16 | 16 |
| KPC-3 |  | dual *mexR*-*oprD* knockout mutant + *bla*_KPC-3_ | *mexAB-oprM* overexpression [↑*mexB* ≈ 10-fold] + OprD deficiency + Carbapenemase | >512 | 16 | >256 | >512 | 16 | >512 | 16 | 8 | 1 | >256 | 32 | >512 | 512 | 16 | 16 |
| VIM-1 | B | dual *mexR*-*oprD* knockout mutant + *bla*_VIM-1_ | *mexAB-oprM* overexpression [↑*mexB* ≈ 10-fold] + OprD deficiency + Carbapenemase | 512 | 512 | >256 | 16 | 16 | >512 | 512 | 8 | 4 | 256 | 256 | 256 | 128 | 128 | 128 |
| IMP-28 |  | dual *mexR*-*oprD* knockout mutant + *bla*_IMP-28_ | *mexAB-oprM* overexpression [↑*mexB* ≈ 10-fold] + OprD deficiency + Carbapenemase | >512 | >512 | >256 | 16 | 16 | 512 | 512 | 8 | 0.5 | 64 | 64 | 256 | 256 | 128 | 256 |
| NDM-1 |  | dual *mexR*-*oprD* knockout mutant + *bla*_NDM-1_ | *mexAB-oprM* overexpression [↑*mexB* ≈ 10-fold] + OprD deficiency + Carbapenemase | >512 | >512 | >256 | 16 | 16 | >512 | 512 | 8 | 32 | >256 | 256 | 512 | 512 | 256 | 512 |
| OXA-14 ^c^ | D | dual *mexR*-*oprD* knockout mutant + *bla*_OXA-14_ | *mexAB-oprM* overexpression [↑*mexB* ≈ 10-fold] + OprD deficiency + ESBL | 512 | 256 | 64 | 32 | 32 | 128 | 16 | 16 | 4 | 16 | 4 | 32 | 32 | 16 | 16 |
| OXA-15 ^d^ |  | dual *mexR*-*oprD* knockout mutant + *bla*_OXA-15_ | *mexAB-oprM* overexpression [↑*mexB* ≈ 10-fold] + OprD deficiency + ESBL | 256 | 256 | 256 | 16 | 16 | 32 | 16 | 8 | 8 | 32 | 4 | 32 | 16 | 16 | 16 |
| CAZ: ceftazidime; C/A: ceftazidime/avibactam; C/T: ceftolozane/tazobactam; ATM: aztreonam; A/A: aztreonam/avibactam; FEP: cefepime; F/T: cefepime/taniborbactam; F/Z: cefepime/zidebactam; FDC: cefiderocol; IMP: imipenem; I/R: imipenem/relebactam; MEM: meropenem; M/V: meropenem/vaborbactam; M/N: meropenem/nacubactam; M/X: meropenem/xeruborbactam.  ^a^ EUCAST v 14.0 breakpoints indicated.  ^b^ Expression levels were calculated relative to PAO1 in previous work.^15–18^  ^c^ OXA-14 is a G157D variant of OXA-10.  ^d^ OXA-15 is a G149R variant of OXA-2. | | | | | | | | | | | | | | | | | | |
